# Positive effects of the enhanced recovery after surgery (ERAS) protocol in DIEP flap breast reconstruction

**DOI:** 10.1016/j.breast.2021.08.010

**Published:** 2021-08-20

**Authors:** N. Gort, B.G.I. van Gaal, H.J.P. Tielemans, D.J.O. Ulrich, S. Hummelink

**Affiliations:** a Radboud University Medical Center, Dept. of Plastic Surgery, Nijmegen, the Netherlands; b HAN University of Applied Sciences, School of Health Studies, Nijmegen, the Netherlands; c Radboud University Medical Center, Radboud Institute for Health Science, IQ Healthcare, Nijmegen, the Netherlands

**Keywords:** Autologous breast reconstruction, Free flap, DIEP flap, Enhanced recovery after surgery, ERAS, Microsurgery

## Abstract

**Background:**

Enhanced recovery after surgery protocols are successfully implemented in different surgical specialties, but a specific protocol for autologous breast reconstruction is missing. The aim of this study was to determine whether an enhanced recovery after surgery (ERAS) protocol contributes to a reduced length of stay without an increase in postoperative complications for patients undergoing a DIEP flap breast reconstruction.

**Materials en methods:**

The effect of the ERAS protocol was examined using a single-center patient-control study comparing two groups of patients. Patients who underwent surgery between November 2017 and November 2018 using the ERAS protocol were compared with a historical control group (pre-ERAS) who underwent surgery between November 2016 and November 2017. The primary outcome measure was hospital length of stay. Secondary outcome measures were postoperative pain and postoperative complications.

**Results:**

152 patients were included (ERAS group, n = 73; control group, n = 79). Mean hospital length of stay was significantly shorter in the ERAS group than in the control group (5 vs. 6 days, p < 0.001). The average pain score was 1.73 in de the ERAS group compared to 2.17 in the control group (p = 0.032). There were no significant differences between the groups in postoperative complications. The ERAS group experienced less constipation (41 vs. 25 patients, p = 0.028).

**Conclusion:**

An enhanced recovery after surgery protocol contributes an accelerated postoperative recovery of patients undergoing a DIEP flap breast reconstruction. In this study a significant decrease was found in hospital length of stay, patient-reported pain score and adverse health issues.

## Highlights

• An (ERAS) protocol can be used to standardize autologous breast reconstruction care.
• Hospital length of stay after a DIEP flap breast reconstruction significantly decreased.
• The ERAS protocol contributes an accelerated postoperative recovery after DIEP breast reconstruction .
• Lower average pain scores and less adverse health issues occurred in patients who were introduced in the ERAS protocol.

## 1 Introduction

Post-mastectomy breast reconstruction has been shown to increase patient's quality of life [[1], [2], [3]]. Breast reconstruction can be performed via implants or autologous tissue transplantation. Amongst the options of autologous breast reconstruction the Deep Inferior Epigastric Perforator (DIEP) flap has become the golden standard nowadays [4,5].

Compared to an implant-based breast reconstruction an autologous reconstruction involves a more complex procedure results in more operative time, a higher morbidity rate and a longer postoperative inpatient monitoring [6]. Given the complexity of the operation intensive pre-, intra- and postoperative care is mandatory to increase the chances of survival of the free flap and the esthetic outcome. For example, it is essential to have a clear preoperative education, perioperative pain management, postoperative flap monitoring and mobilization of the patient [4]. Although the DIEP flap became the golden standard worldwide, there is not yet an uniform protocol for the pre-, intra- and postoperative care [6].

A way of standardizing autologous breast reconstruction care is to use an enhanced recovery after surgery protocol. Enhanced recovery after surgery (ERAS) protocols were successfully implemented in many procedures in different surgical specialties [7,8]. The ERAS protocol consists of standardized pre-, intra- and postoperative care to improve postoperative recovery and outcomes, leading to improved mobility and shorter length of stay without an increase in complications [6,9,10].

Given the importance for standard care and a clear protocol for the DIEP breast reconstruction procedure a postoperative ERAS protocol was implemented in our university hospital. Perioperative anesthesia and pain management were already standardized and remained unaltered in this ERAS protocol. Before implementation of the ERAS protocol, patients’ expectations were insufficiently managed and health care providers followed unstandardized protocols for the postoperative care. Due to the severity and complexity of the surgery and lack of standardization of postoperative care, the hospital length of stay varies from four to seven days. The implementation of a default postoperative ERAS protocol based on previous studies, gave us the opportunity to evaluate the effect of this standardization of care.

The aim of this study was therefore to determine whether an enhanced recovery after surgery (ERAS) protocol contributes to a reduced length of stay without an increase in postoperative complications for patients undergoing a DIEP flap breast reconstruction.

## 2 Materials and methods

A patient-controlled study was established to examine of the hospital records of patients who underwent a DIEP flap in our teaching hospital. Patients were included if they underwent an immediate or delayed, unilateral or bilateral DIEP flap breast reconstruction. Only patients who simultaneously underwent a prophylactic ovariectomy were excluded. An analysis of the collected data was performed to compare patients using the enhanced recovery after surgery protocol (operated between November 2017 and November 2018) an those before the introduction of the enhanced recovery after surgery protocol (operated between November 2016 and November 2017). All operations had been performed by experienced plastic surgeons and residents.

The ERAS protocol focused on improved preoperative information and standardization of postoperative care for patients undergoing a DIEP flap breast reconstruction, whereas the intraoperative surgical procedure and intra- and postoperative pain management remained unchanged. All patients received intravenous pain medication during surgery. After recovering in the post-anesthesia unit patients were transferred to the plastic surgery ward were they received scheduled administration of paracetamol and diclofenac. In addition patients got a Patient Controlled Analgesia (PCA) pump with Morphine or Piritramide the first operative days. After removal of the intravenous line and PCA pump, oral opioids were available to patients if the pain score was higher than four, measured using the Numeric Rating Scale (NRS) [11]. Closed suction drains were placed on both sides of the abdomen and in the reconstructed breast. Closed suction drains were removed when the production was less than 30 ml per 24 h or after a maximum of 14 days. Having a closed suction drain does not affect the patient's hospital length of stay.

Standardization of postoperative care via the ERAS protocol (Table 1) consisted of encouraged mobilization from de first day after surgery (day 1), removal of the bladder catheter and removal of intravenous line and PCA pump (day 2), independent mobilization at day 3 and discharge from hospital at day 4.

**Table 1:** The ERAS protocol.

Preoperatively	Day of surgery (after surgery)	POD 1	POD 2	POD 3	POD 4 (Goal discharge day)
Information about standardization of postoperative care	Bedrest, lie on back, hips and knees flexed	Mobilization up to a chair or bedside (with abdominal binder)	Mobilization at least 3 times a day up to a chair (with abdominal binder)	Mobilization at least 4 times a day up to a chair or walking around (with abdominal binder)	Mobilization at least 6 times a day up to a chair or walking around (with abdominal binder)
	Checkup of the flap every hour	Checkup of the flap every hour	Checkup of the flap every 2 h	Checkup of the flap 3 times a day	Checkup of the flap 3 times a day
	IV line + Antibiotics	IV line + Antibiotics	Removal of the IV line		
	Bladder catheter	Bladder catheter	Removal of bladder catheter		
	Pain medication: Paracetamol, diclofenac and PCA	Pain medication: Paracetamol, diclofenac and PCA	Pain medication: Paracetamol, diclofenac	Pain medication: Paracetamol, diclofenac	Pain medication: Paracetamol, diclofenac
	Vacuum drains (removed when output <30 cc/24 h)	Vacuum drains (removed when output <30 cc/24 h)	Vacuum drains (removed when output <30 cc/24 h)	Vacuum drains (removed when output <30 cc/24 h)	Vacuum drains (removed when output <30 cc/24 h)
	Regular diet (energy and protein enriched)	Regular diet (energy and protein enriched)	Regular diet (energy and protein enriched)	Regular diet (energy and protein enriched)	Regular diet (energy and protein enriched)
		Physiotherapy - Respiratory exercises	Respiratory exercises	Respiratory exercises	Respiratory exercises
					Compression bra

Abbreviations: POD, postoperative day, PCA, patient controlled analgesia.

All patients received a patient version of this protocol, in which the postoperative care was described in forms of goals that patients are expected to achieve per day during hospital admission. This ERAS protocol was implemented in October 2017. The pre-ERAS protocol consisted of a guideline based on available literature and adjusted in collaboration with plastic surgeons and nurses with which patients were discharged from hospital after 5–7 days depending on the degree of mobilization and postoperative recovery [12].

The primary outcome measure was hospital length of stay, defined as the number of admitted days from the day of surgery (postoperative day 0) to the day of discharge.

Secondary outcome measures were postoperative pain and incidence of postoperative complications. Postoperative pain was measured using the Numeric Rating Scale (NRS). With the NRS pain can be rated on a scale from 0 (no pain) to 10 (worst pain). The NRS was scored three times a day during admission [11].

Postoperative complications related to surgery were defined as those occurring in the early stage from surgery until hospital discharge (complete or partial flap loss, major bleeding, necrosis and pneumothorax) and in the late stage, from hospital discharge up to 14 postoperative days (necrosis, wound dehiscence, surgical site infection and seroma). Major bleeding, wound dehiscence and seroma were only recorded if a reoperation or puncture was needed. A surgical site infection was only recorded if treated with antibiotics.

In addition, adverse health events during hospital admission were recorded, included constipation, pneumonia, pulmonary embolism, deep vein thrombosis and urinary tract infection.

Patient characteristics, preoperative and perioperative data, such as age, body mass index (BMI), American Society of Anaesthesiologists (ASA) and comorbidity were collected to compare between the ERAS group and the control group.

SPSS statistics version 22 (IBM, Armonk, NY, USA) was used for all statistical analyses. Depending on skewness, the descriptive statistics were reported as number of patients with percentages, mean and SD or median and interquartile range. The difference between de ERAS and control group in continuous variables hospital length of stay and postoperative pain were analyzed using the Student's independent *t*-test. The difference in number of complications between the groups were analyzed using the chi-squared or Fisher's exact test. Multivariate regression analysis was used to evaluate the difference between the two groups on the primary and secondary outcomes adjusted for BMI and ASA classification. A Poisson regression analysis was done to analyze pain score and account for risk factors as BMI and ASA classification. A value of p < 0.05 was considered statistically significant.

## 3 Results

A total of 152 patients were included in this study. The ERAS group consisted of 73 patients who underwent a DIEP flap reconstruction according to the enhanced recovery after surgery protocol. The control group consisted of 79 patients. One patient was excluded due to metastatic disease diagnosed shortly after surgery. Patient characteristics, preoperative and operative details are presented in Table 2.

**Table 2:** Patient characteristics, preoperative and operative details.

Characteristic	Patient group		p-value
	Pre-ERAS	ERAS	
Patients	n = 79	n = 73	
Age, yr (SD)	50.1 (9.12)	51.1 (9,36)	0.497
BMI, kg/m^2^ (SD)	27.1 (2.32)	26.3 (2.46)	0.041^b^
ASA score, n (%)			0.294^c^
I	27 (34)	19 (26)	
II	50 (63)	52 (71)	
III	2 (3)	2 (3)	
Active smoker, n (%)	0 (0)	2 (3)	0.139
Comorbidity, n (%)	22 (28)	24 (33)	0.500
Hypertension	7 (9)	5 (7)	
Diabetes Mellitus	3 (4)	5 (7)	
Asthma	6 (8)	7 (10)	
COPD	1 (1)	2 (3)	
Crohn's disease/Ulcerative Colitis	2 (3)	0 (0)	
Other	8 (10)	8 (11)	
Chest wall radiation, n (%)	39 (49)	40 (55)	0.503
Timing of reconstruction, n (%)			0.542
Immediate	16 (20)	10 (14)	
Delayed	56 (71)	55 (75)	
Both^a^	7 (9)	8 (11)	
Laterality, n (%)			0.951
Unilateral	48 (61)	44 (60)	
Bilateral	31 (39)	29 (40)	

a Includes patients who had bilateral breast reconstruction with 1 side immediate and the other delayed.

b Statistical significant.

c Included in the multivariate regression analysis; Abbreviations: BMI, Body Mass Index kg/m [2], ASA, American Society of Anesthesiologist Physical Status, COPD, Chronic Obstructive Pulmonary Disease.

Patients in the ERAS group had a statistical significant lower BMI (p = 0.041) and less patients with ASA score I were in the control group.

Hospital length of stay was one day shorter in the ERAS group compared to the pre-ERAS group (5 days versus 6 days; p < 0.001). No significant difference was found in hospital length of stay in distinguishing unilateral or bilateral surgery and primary and secondary surgery.

The average pain score during admission was 1.73 in the ERAS group compared to 2.17 in the control group, which was statistical significantly lower (p = 0.032). The postoperative outcomes are shown in Table 3.

**Table 3:** Postoperative outcomes.

Outcome	Patient group		Difference	p-value	95% CI
	Pre-ERAS (n = 79)	ERAS (n = 73)			
Hospital LOS	6.2 (1.31)	5.0 (1.20)	1.14	<0.001^a^	[0.73 … 1.54]
Postoperative pain	2.17 (1.32)	1.73 (1.35)	0.44	0.032^a^	[0.04 … 0.83]
Morning	2.15 (1.32)	1.79 (1.30)	0.35	0.110	[-0.08 … 0.79]
Afternoon	2.19 (1.50)	1.94 (1.38)	0.25	0.273	[-0.20 … 0.71]
Evening	2.16 (1.48)	1.46 (1.13)	0.70	0.001^a^	[0.28 … 1.13]

Values are mean (SD).

Abbreviations: CI, confidence interval, LOS, length of stay.

a Statistical significant.

A comparison of postoperative complications between the ERAS group and the control group is shown in Table 4. There were no statistical significant differences in early or late complication rate between the ERAS group and the control group. More than half of the patients (52%) in the pre-ERAS group had constipation compared to one third of the patients (34%) in the ERAS group (42 versus 26 patients), which was statistically significant (p = 0.028).

**Table 4:** Postoperative complications.

Complication	Patient group		p-value
	Pre-ERAS	ERAS	
Patients	79	73	
Early complications, from surgery to discharge n (%)			
Partial flap loss	1 (1.3)	0 (0)	1.000
Complete flap loss	4 (5.1)	2 (2.7)	0.683
Major bleeding	4 (5.1)	2 (2.7)	0.683
Donor site necrosis	0 (0)	1 (1.4)	0.480
Pneumothorax	1 (1.3)	0 (0)	1.000
Late complications, from discharge to 2 weeks n (%)			
Necrosis	1 (1.3)	3 (4.1)	0.351
Surgical site infection	3 (3.8)	3 (4.1)	1.000
Wound dehiscence	1 (1.3)	1 (1.4)	1.000
Seroma	3 (3.8)	4 (5.5)	0.711
Adverse health issues, n (%)			
DVT	0 (0)	0 (0)	–
Pulmonary embolism	0 (0)	0 (0)	–
Pneumonia	1 (1.3)	1 (1.4)	1.000
Urinary tract infection	1 (1.3)	0 (0)	1.000
Constipation	41 (52)	25 (34)	0.028^a^

a Statistical significant; Abbreviations: DVT, deep vein thrombosis.

## 4 Discussion

In this study it was determined whether an enhanced recovery after surgery (ERAS) protocol contributes to an accelerated postoperative recovery for patients undergoing a DIEP flap breast reconstruction, which means a reduced length of stay without an increase in postoperative pain or postoperative complications. After implementing the postoperative ERAS protocol, hospital length of stay decreased from 6 to 5 days, a significant lower average pain score was recorded and less constipation occurred in the ERAS group. There was no significant difference between the two groups in number of complications.

Throughout literature, various length of stay have been reported in DIEP flap breast reconstructions due to the heterogenicity of the patients en the differences in health care systems. However, all studies report a decrease in length of hospital stay after implementing an enhanced recovery after surgery protocol [[13], [14], [15]]. This study shows a decrease in length of stay from 6 to 5 days which is similar to a decrease of one or two days shown in studies by Rochlin et al. (2019), Astanehe et al. (2018), Afonso et al. (2017) and Batdorf et al. (2014).

Previous studies report lower patient reported pain scores after the implementation of an ERAS protocol, although the method of measurement differs between the studies. Our presented study measured an average pain score in the morning, afternoon and evening, where a significant decrease of average pain in the evening in the ERAS group was seen (2.19 versus 1.46; p = <0.001). Furthermore, a significant decrease of average pain during admission was seen in our presented study (2.17 versus 1.73; p = 0.032), which is comparable to results as reported by Astanehe et al. [16] Afonso et al. (2017) [17] and Batdorf et al. (2014) [18] reports significant lower pain scores, however they measured a pain score every four to 6 h, meaning the time of the day is variable. Also Sharif-Askary et al. (2019) [19] has shown lower patient reported pain scores, although not significant. In this study, postoperative pain management remained unchanged, where as other studies changed the way pain was reduced [[16], [17], [18],^20^]. This favors the idea that a decrease in pain scores is experienced when patients are introduced to the ERAS protocol.

The enhanced recovery protocol did not lead to a significant increase in complication rate and the occurrence of complications in this study was found to be comparable to the other studies [[16], [17], [18], [19], [20], [21], [22]]. However, the number of patients with a wound infection or wound dehiscence turned out to be lower in this study compared to the results presented by Kaoutzanis et al. (2018) [20]. In none of the previous studies in breast reconstruction is the occurrence of constipation included in the analysis. In this study, a significantly decrease of number of patients with constipation was seen. This could be the result of early mobilization which can accelerate bowel movements. This is comparable to a study by Li et al. (2018) [23] where they have seen a significant decrease in the time to first bowel after implementing an ERAS protocol in cardiac surgery patients.

Our team of plastic surgeons are experienced in performing DIEP flap breast reconstruction surgeries, and have been operating on both the ERAS as pre-ERAS patient cohorts. Although the study is not a randomized controlled trial, the groups in this retrospective cohort study are comparable as no changes in staff or (post)-operative workflow except the ERAS protocol implementation had been performed.

Enhanced recovery after surgery protocols have led to improved mobility and shorter length of stay without an increase in complications in various surgical and orthopedic operations, leading to implementation of ERAS as standard care [[23], [24], [25], [26]]. Similar results have been reported in literature in microsurgical free flap breast reconstructions, although these were not implemented as standard care and protocols vary between different medical centers*.* A reduced length of stay can lead to a reduction in healthcare costs and above all, it can have a positive influence on patients’ satisfaction and quality of life [13]. These factors were not included in this study but could be investigated in a follow-up study.

As no increase in early or late complications has been observed, and the pain score decreased significantly, we believe that patients can be safely discharged from the hospital one day earlier following the ERAS protocol. Following the results of this study, the ERAS protocol is nowadays utilized and implemented by default for breast reconstructive surgery in our hospital.

## 5 Conclusion

The introduction of an enhanced recovery after surgery protocol contributes an accelerated postoperative recovery of patients undergoing a DIEP flap breast reconstruction. In this retrospective cohort study a significant decrease was found in hospital length of stay, without an increase of postoperative complications. Moreover, the results of this study demonstrates a significant decrease in patient-reported pain scores and adverse health issues. The ERAS protocol is now embedded in our default clinical practice.

## Funding

This research did not receive any specific grant from funding agencies in the public, commercial, or not-for-profit sectors.

## Declaration of competing interest

None.
